# Phylogenomics resolves the etiology of dieback disease and deciphers *Ceratocystis dalbergicans* sp.nov., causal agent of *Dalbergia sissoo* decline

**DOI:** 10.3389/fgene.2023.1136688

**Published:** 2023-03-14

**Authors:** Imran Ul Haq, Siddra Ijaz, Muhammad Zunair Latif, Iqrar Ahmad Khan, Hayssam M. Ali, Sukhwinder Kaur

**Affiliations:** ^1^ Department of Plant Pathology, The University of Agriculture Faisalabad, Faisalabad, Pakistan; ^2^ Centre of Agricultural Biochemistry and Biotechnology, The University of Agriculture Faisalabad, Faisalabad, Pakistan; ^3^ Institute of Horticultural Sciences, The University of Agriculture Faisalabad, Faisalabad, Pakistan; ^4^ Department of Botany and Microbiology, College of Science, King Saud University, Riyadh, Saudi Arabia; ^5^ Department of Plant Pathology, University of California, Davis, CA, United States

**Keywords:** novel species, fungi, dieback, Ceratocystis, *Dalbergia sissoo*, conservation

## Abstract

*Dalbergia sissoo* is one of the most economically important trees in forestry, agroforestry, and horticulture. This tree species is severely threatened by dieback. Widespread dieback outbreaks and infestations have drastically destroyed billions of *D. sissoo* trees. Hence, we attempted to resolve the dieback etiology through phylogenomics associated with *D. sissoo* mortality. The Ceratocystis species was evaluated using morphologically investigated fungal isolates collected from dieback-affected tissue plants. Based on the symptomatology, we have differentiated dieback from Fusarium wilt and concluded that the Ceratocystis fimbriata sensu lato complex is causing shisham dieback in Pakistan. As the Ceratocystis species complex is a cryptic species complex, we used genomics and phylogenetic analysis for deciphering its evolutionary hierarchical order. The pathogen’s operational taxonomy was unlocked with the help of phylogenomics, and it was discovered that isolates from *D. sissoo* represent a species distinct from the other species in the *C. fimbriata* sensu lato species complex. The name *Ceratocystis dalbergicans* sp. nov. has been given to the fungus causing dieback disease in *D. sissoo*.

## 1 Introduction

*Dalbergia sissoo* (Shisham) is a member of the eukaryotic kingdom Plantae, phylum Spermatophyta, subphylum Angiospermae, class Dicotyledonae, order Fabales, family Fabaceae, subfamily Faboideae, and genus Dalbergia (Ijaz et al., 2021a). It is a moisture-loving, fast-growing, medium-to large-sized deciduous tree that grows best on alluvial soils at a pH between 5.0 and 7.7 (Kumar and Khurana, 2016; Ijaz et al., 2021b). It is one of the trees with the greatest economic value in forestry, agroforestry, and horticulture (Ijaz et al., 2018). In addition to its commercial importance, *D. sissoo* exhibits medicinal and industrial uses: it is a source of fuel, alkaloids, fibers, neoflavonoids, resins, and tannins (Ijaz et al., 2021a), and is also used as a landscaping tree (Kumar and Khurana, 2016; Ijaz and Ul Haq, 2021).

Dieback is a commonly found disease in *D. sissoo* in Bangladesh, India, Nepal, and Pakistan (Ijaz et al., 2019; Naqvi et al., 2019; Ul Haq et al., 2021). In the past 100 years, widespread dieback outbreaks and infestations have drastically reduced *D. sissoo* densities and killed billions of *D. sissoo* trees (Webb and Hossain, 2005; Rehman et al., 2012; Naqvi et al., 2019; Ul Haq et al., 2021). Symptoms of D. sissoo dieback disease include yellowing, drying, and defoliation of leaves and branches; partial or complete wilting of the crown leading to yellowing of the whole plant; thinning of leaves and crown; internal stem and root browning; chlorosis; necrosis; top dieback; dieback of branches at initial and lateral stages; gummosis; vascular discoloration; and bark splitting (Poussio et al., 2010; Mukhtar et al., 2014b; Latif et al., 2021). Researchers have made several attempts to find out the pathological cause of the disease since its identification. They have documented many fungal pathogens that cause dieback in *D. sissoo* and proposed management strategies. Despite a long history of pathological research studies on *D. sissoo* dieback, the infection persists to this day in *D. sissoo* plantations with a varying frequency of incidence and severity. This indicates that further research is needed to explore the pathological cause of the disease by employing conventional and molecular pathological techniques (Ijaz et al., 2020). Numerous studies attempted to elucidate the biology of *D. sissoo* dieback (Hassan et al., 2021). Several fungal pathogens, including Botryodiplodia theobromae, Fusarium spp., Ganoderma lucidum, Phytophthora cinnamomi, and Ceratocystis, have been linked to dieback and mortality on *D. sissoo* plantations. Although some researchers have discussed managing *D. sissoo* dieback in the Asian subcontinent by using host resistance genes or chemical control, their work has focused primarily on F. solani as the causal agent (Dayaram et al., 2003; Javaid, 2008; Ul Haq and Ijaz, 2020). However, C. fimbriata was reported a decade ago as a leading cause of *D. sissoo* mortality in Pakistan (Al Adawi et al., 2009; Poussio et al., 2010; Ul Haq et al., 2019). There is a clear association between Ceratocystis and dieback in *D. sissoo*, although the specific pathogen that causes dieback in *D. sissoo* has not been determined. Identifying the etiology and overcoming the disease has proven challenging and needs to be addressed. Hence, we completed Koch’s postulates with isolates of Fusarium solani and the Ceratocystis fimbriata sensu lato species complex (Ul Haq et al., 2019) from symptomatic *D. sissoo* sampled from 117 sites across Pakistan. We observed the distinctive symptoms. Based on the symptomology, we have differentiated the dieback from Fusarium wilt and concluded that a species of Ceratocystis fimbriata sensu lato complex is causing shisham dieback in Pakistan.

This research provided a scientific understanding of *D. sissoo* dieback disease by focusing on the emerging pathogen, Ceratocystis, associated with *D. sissoo* mortality. This research study was conducted under CAS-PARB Project No. 952. In this study, the etiology of dieback was resolved through phylogenomics. Seven genetic loci were sequenced for phylogenetic analyses to decipher hierarchy, including the internal transcribed spacer (ITS), the translation elongation factor1-alpha (TEF1-α), the second-largest subunit of RNA polymerase II (RPBII), calmodulin (CAL), the guanine nucleotide-binding protein subunit beta-like protein (MS204), a subunit of mini-chromosome maintenance proteins (MCM7), and tubulin (β-tubulin). The Ceratocystis isolates were discovered by phylogenomics analysis to be a novel species in the Ceratocystis species complex that causes dieback disease in *D. sissoo*.

## 2 Materials and methods

### 2.1 Genomic analysis for unlocking the Ceratocystis taxonomy

#### 2.1.1 DNA extraction

We isolated the DNA from mycelial cultures of Ceratocystis isolates using a genomic DNA extraction kit GeneJET Genomic DNA Purification Kit (Thermo Fisher Scientific, USA) and monitored the integrity of the extracted DNA through agarose gel electrophoresis. The isolated DNA samples were quantified at an absorbance of 260/280 nm by UV-visible NANODROP (8,000 Spectrophotometer, Thermo Fisher Scientific).

#### 2.1.2 PCR analysis

We explored the taxonomy of Ceratocystis isolates by unlocking the seven genetic loci: the internal transcribed spacer (ITS), the translation elongation factor 1-alpha (TEF1-α), the second-largest subunit of RNA polymerase II (RPBII), calmodulin (CAL), the guanine nucleotide-binding protein subunit beta-like protein (MS204), a subunit of minichromosome maintenance proteins (MCM7), and tubulin (β-tubulin) (Table 1). The partial region amplification of these loci using their respective sequence-specific primer pairs in the PCR analysis was carried out in the VeritiTM 96-Well Fast Thermal Cycler by Applied Biosystems. The PCR products were electrophoresed through high-resolution agarose (ACTGene, USA), eluted using the FavorPrep Gel Purification Kit (Favorgen Biotech Corporation, Taiwan), and then cloned into pTZ57R/T (InsTAcloneTM PCR cloning kit).

**TABLE 1:** Primers used for phylogenetic analysis at different loci (ITS, TEF1-α, RPBII, CAL, MS204, MCM7, and β-tubulin).

Sr#	Gene region	Primer pair	References
1	*ITS*	ITS1-F/ITS4	White et al. (1990)
2	TEF-CER	EFCF1/EFCF6	Harrington (2009)
3	*cal*	CAL2F/CAL2R2	Duong et al. (2012)
4	*β-tub*	βt1a/βt1b	Glass and Donaldson (1995)
5	RPBII	RPB2-5Fb/RPB2-7Rb	Fourie et al. (2015)
6	MS204	MS204F.ceratoB/MS204R.ceratoB	Fourie et al. (2015)
7	MCM7	Mcm7-709/Mcm7-1,348	Schmitt et al. (2009)

#### 2.1.3 DNA sequencing

The sequencing of the cloned fragments was executed bidirectionally by Eurofins Genomics DNA sequencing services, United States. High-quality (HQ) sequences were obtained using the sequence alignment editor, BioEdit version 7.2.6. The DNASTAR Lasergene v. 7.1.0 SeqMan Pro (SeqManTMII) software package was used for generating consensus sequences before their deposit in GenBank to get accession numbers.

### 2.2 Phylogenomic studies

#### 2.2.1 Phylogenetic analysis

For phylogenetic comparison, we supplemented the generated sequences of seven genic regions with available sequences from the NCBI database, based on their homology and literature (Supplementary Table S1). The ClustalW program was used to perform multiple sequence alignments (MSAs) of each individual dataset. The concatenated dataset from the aligned data of individual partitions was generated with Geneious software version 4.8.5, which was also used to check the quality of the alignments. Alignments were deposited in the nexus file in TreeBASE, which was generated using Mesquite version 3.51. In a phylogenetic analysis of a concatenated dataset of seven loci, Maximum Parsimony (MP) analysis using PAUP* version 4.0a161 software was employed for tree construction. Maximum-parsimony genealogies were constructed by selecting the bootstrap method in the heuristic search with 1,000 replicates, random stepwise addition for ten replicates, tree-bisection-reconstruction (TBR) as the branch-swapping algorithm with a reconnection limit (8), branches collapsing when the maximum branch length was zero, gaps being treated as a new state (5th base) with the “MulTrees” option in effect, and characters being of unord-type with equal weight. Different metrics, including tree length (TL), homoplasmy index, consistency index (CI), rescaled consistency index (RC), and retention index (RI), were also computed in the MP analysis.

#### 2.2.2 Coalescent species tree method

For validating the concatenated dataset-based phylogenetic analysis, a coalescent species tree approach was implemented to construct a species tree by employing *BEAST software version 2.5.0. The BEAST (Bayesian Evolutionary Analysis for Sampling Tree) input files were generated through the BEAUti (Bayesian Evolutionary Analysis Utility) program under a non-standard template, StarBEAST, to set up an evolutionary model and an MCMC run to generate XML files importing aligned individual dataset files (input files) in NEX format. A linear and-constant root model was implemented as a multi-species coalescent model; however, Yule model selection as species tree priors was made to specify speciation time distribution. The maximum clade credibility species tree was constructed by TreeAnnotator version 2.3.0 with 10% burin and a 0.5 posterior probability limit to identify a tree that denotes the best posterior distribution. The Figtree software was used to visualize the species tree.

## 3 Results

We performed phylogenomic analysis on nine fungal isolates collected from diseased tissues of dieback-affected *Dalbergia sissoo* plants. These were selected for phylogenetic analysis using the findings of multilocus sequence typing (MLST) analysis of twenty-three isolates morphologically identified as the Ceratocystis fimbriata sensu lato species complex, a complex of cryptic species (Ul Haq et al., 2019). The generated sequences of seven loci were lodged in the NCBI database with GenBank accession numbers described in Supplementary Table S1.

**FIGURE 1:**
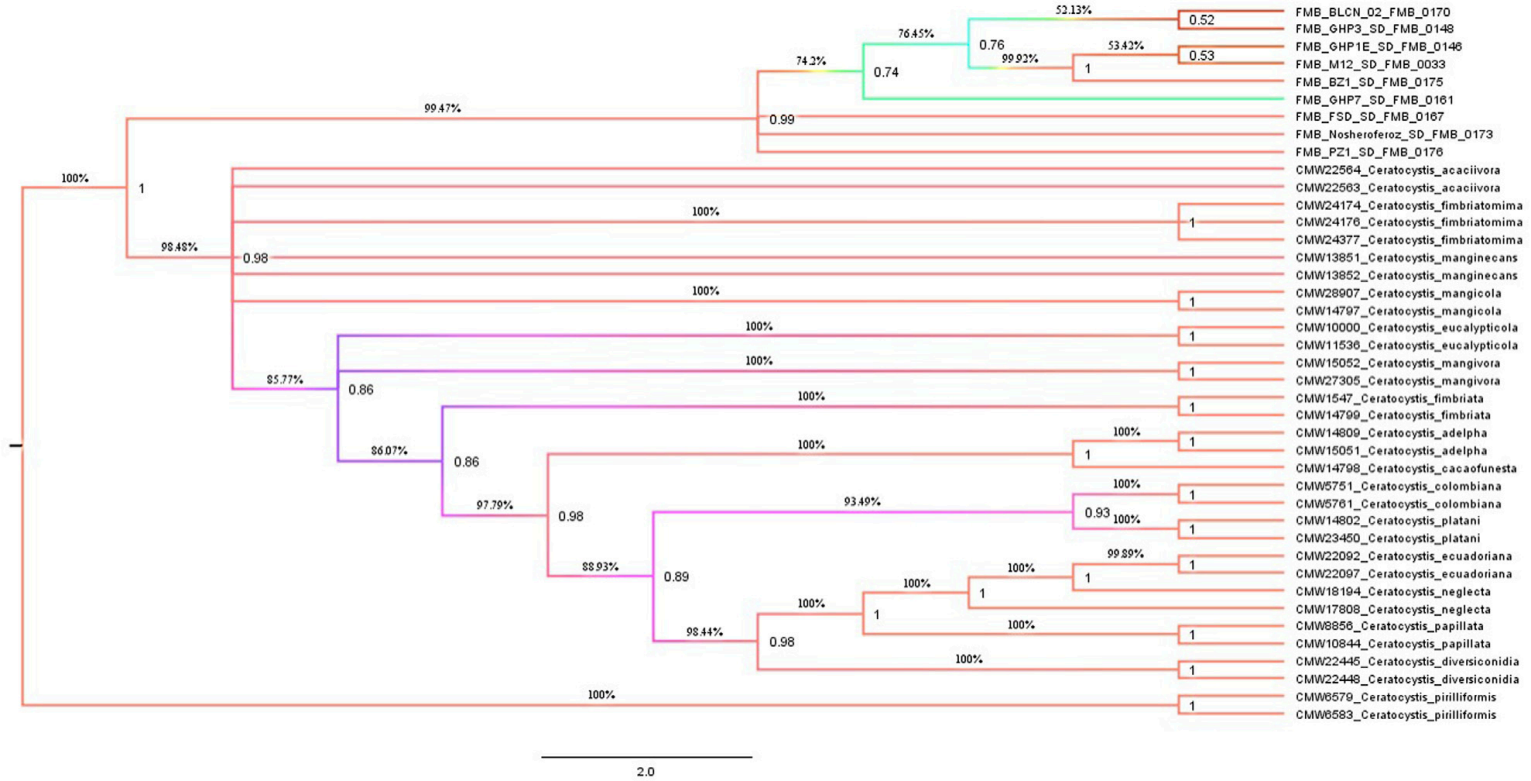
Maximum-parsimony genealogies were constructed by selecting the bootstrap method in heuristic search with 1,000 replicates, random stepwise addition for ten replicates, tree-bisection-reconstruction (TBR) as the branch swapping algorithm with a reconnection limit (8), branches collapsed when maximum branch length was zero, with the “MulTrees” option in effect.

**FIGURE 2:**
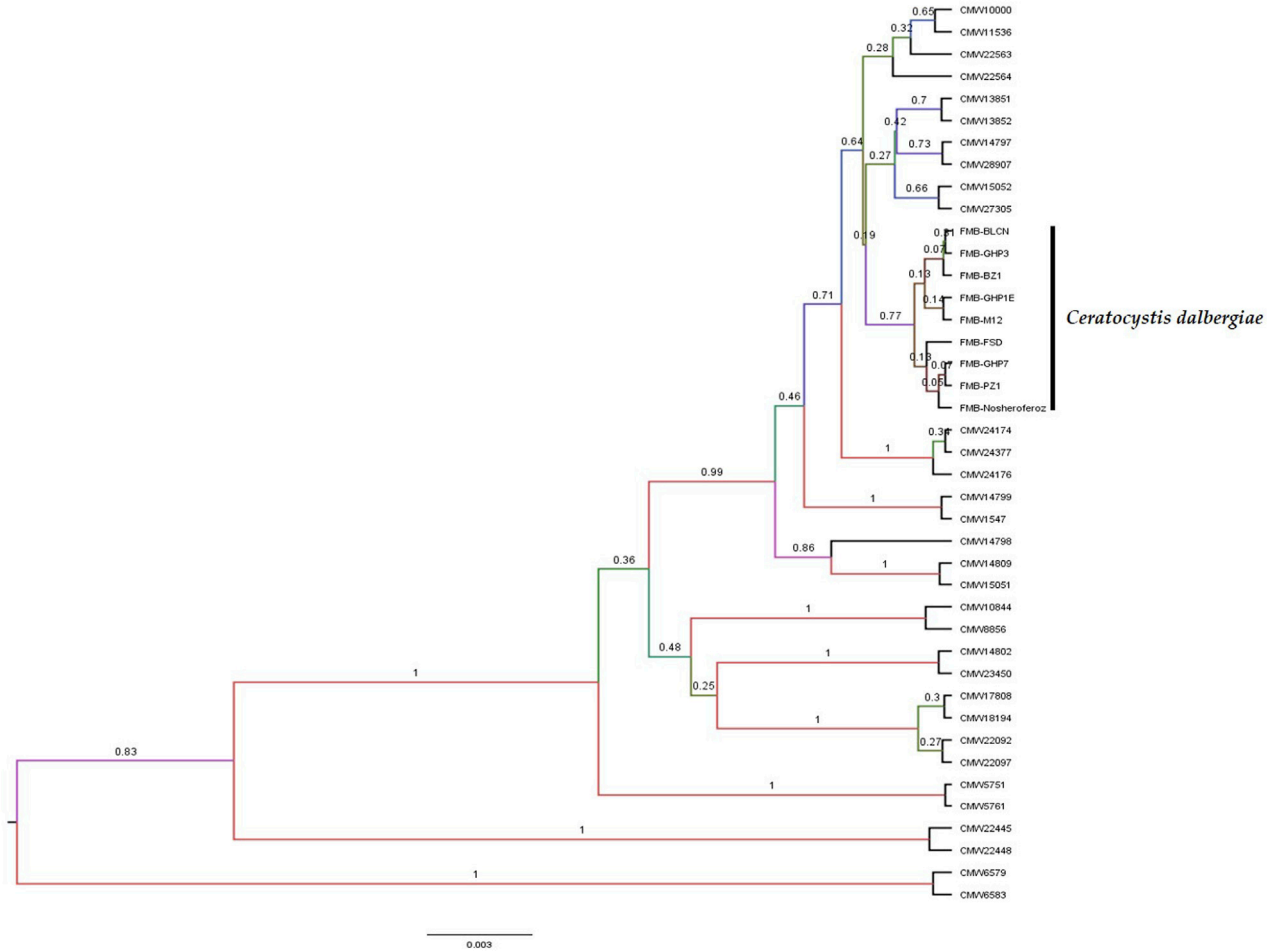
Species tree generated performing a BEAST analysis revealed that Ceratocystis isolates from *Dalbergia sissoo* dieback-affected tissues are more diverse than other Ceratocystis species and have been proven to be a new Ceratocystis species that causes dieback in *Dalbergia sissoo*. The scale bar depicts the expected number of changes per site.

A Maximum Parsimony (MP) analysis was executed on a concatenated dataset of seven loci for estimating species boundaries by generating a maximum parsimonious (MP) tree through PAUP* software. The majority rule bootstrap (50%) consensus tree was based on 3,590 parsimony-informative characters (PIC) with a tree length of 6,723. The computed matrices of this analysis were 0.2829 homoplasmy index (HI), 0.7289 consistency index (CI), 0.8910 rescaled consistency index (RC), and 0.6494 retention index (RI). The MP tree identified the FMB isolates (collected fungal isolates from the diseased tissues of dieback-affected *D. sissoo* plants) as a separate and distinctly diverged clade with 99.47% bootstrap support and a posterior probability (PP) support value of 0.99. A posterior probability value > 0.94 (94%) is considered to be strong support for separate taxa. The 94% speciation probability value is considered strong support for a speciation event (Figure 1). However, validating the MP analysis (Concatenation-based phylogenetic analysis), we employed the coalescent species tree method (Coalescent-based phylogenetic analysis) for the first time in Ceratocystis species delimitation studies. BEAST (Bayesian Evolutionary Analysis for Sampling Tree) analysis was made by executing *BEAST software for species tree generation. The species tree revealed strong concurrence with the most parsimonious phylogenetic tree and displayed clear divergence of these fungal isolates from other Ceratocystis species (Figure 2). Hence, considering the MP tree and species tree, we suggested these isolates as a new Ceratocystis species causing dieback in *D. sissoo* and named them ***Ceratocystis dalbergicans*** sp. nov.

## 4 Taxonomy

***Ceratocystis dalbergicans*** I.U. Haq, S. Ijaz, I. A. Khan and M. Z. Latif, sp. nov; **basionym:** *Ceratocystis dalbergiae;* **MycoBank 841380.** NCBI:txid2870506.

**Material examined: Pakistan**, from living stems, and branches of the shisham tree, February and March 2017 and 2018, I.U. Haq (FMB H 13.1, Holotype, ex-type culture FMBCC 13.1).

Note: The strain is preserved in a metabolically inactive state.

*Etymology:* Named after the host (Shisham, in English; *Dalbergia sissoo*, botanical name) from which these were first isolated.

## 5 Discussion

*Dalbergia sissoo* dieback disease is a national issue in Pakistan and other South Asian countries (Dayaram et al., 2003; Webb and Hossain, 2005; Shakya and Lakhey, 2007; Mukhtar et al., 2014a). No data exist that concern the distribution, prevalence of dieback disease, and losses associated with *Ceratocystis* in Pakistan. However, reports are available on other fungal pathogens from limited areas of Punjab or Sindh province, which do not represent the overall situation of this issue (Bajwa et al., 2003; Khan et al., 2004; Rajput et al., 2010).

According to their morphology, the fungal isolates from dieback-affected *D. sissoo* trees were identified as possible *Ceratocystis fimbriata* sensu lato members (a cryptic species complex). Therefore, these isolates were characterized using primary and secondary DNA barcodes. The ITS region was used as the primary DNA barcode because this region has been used to distinguish species of the Ceratocystis genus (Van Wyk et al., 2009). However, two ITS types were identified *Ceratocystis mangenicans* suggested attentiveness while using this region for species delineation in the Ceratocystis species complex (Al Adawi et al., 2013), despite a phylogeny based on a combined dataset including TEF1-α and β tubulin strongly supported a speciation event (Tarigan et al., 2011; Al Adawi et al., 2013). Fourie et al. (2015) documented this combined tree gene phylogeny, which showed no well-supported species discrimination in the case of other Ceratocystis species and suggested βt1, MS204, and RPB11 as strong markers in combination to delineate the Ceratocystis species complex. Therefore, we used seven genetic loci: the internal transcribed spacer (ITS), the translation elongation factor1-alpha (TEF1-α), the second-largest subunit of RNA polymerase II (RPBII), calmodulin (CAL), guanine nucleotide-binding protein subunit beta-like protein (MS204), a subunit of mini-chromosome maintenance proteins (MCM7), and tubulin (β-tubulin). The combined seven gene phylogeny revealed support for species delineation for these Ceratocystis isolates as a new taxon of this complex.

Data presented in this study supports the hypothesis that *Ceratocystis* sp. is the primary fungal pathogen responsible for Pakistan’s widespread *D. sissoo* dieback disease. Our research findings are supported by other studies that conclude *D. sissoo* mortality in Pakistan is due to *Ceratocystis* sp. (Al Adawi et al., 2009; Poussio et al., 2010; Ul Haq et al., 2019). However, other scientists claimed that *Botryodiplodia theobromae* (Khan et al., 2004; Rehman et al., 2012), *Fusarium* spp. (Dayaram et al., 2003; Shakya and Lakhey, 2007; Rajput et al., 2008), *Ganoderma lucidum* (Parker, 1915; Troup, 1921; Sharma et al., 2000), and *Phytophthora cinnamomi* (Gill et al., 2002; Rehman et al., 2012) caused *D. sissoo* dieback. On the contrary, we have established that the novel species *C. dalbergicans* is the primary cause of dieback disease in *D. sissoo* in Pakistan.

## Data Availability

The datasets presented in this study can be found in online repositories. The names of the repository/repositories and accession number(s) can be found in the article/Supplementary Material.
