# Supplementary material for: Phylogenomics resolves the etiology of dieback disease and deciphers Ceratocystis dalbergicans sp.nov., causal agent of Dalbergia sissoo decline
Source: Front Genet. 2023 Mar 14;14:1136688. doi: 10.3389/fgene.2023.1136688 (PMC10043428; doi:10.3389/fgene.2023.1136688)
Supplement: Supplementary file 1 [file Table1.docx]

**Supplementary Table 1: A list of isolates names, strain accession numbers, and GenBank accession numbers assigned to FMB fungal isolates of the shisham tree by the NCBI database**

| **Isolate/Species name** | **Culture/strain accession number** | **GenBank accession numbers** | | | | | | | **Host** | **Country** |
| --- | --- | --- | --- | --- | --- | --- | --- | --- | --- | --- |
|  |  | **ITS** | **TEF1-α** | **β-tubulin** | **MS204** | **RPBII** | **MCM7** | **CAL** |  |  |
| FMB-M12-SD | FMB 0033 | MK890321 | MF767443 | OQ200442 | OQ332716 | MW287596 | MW928519 | OQ348108 | *Dalbergia sissoo* | Punjab, Pakistan |
| FMB-GHP1E-SD | FMB 0146 | MK890310 | MK816923 | MZ397950 | OQ332717 | MW296053 | MW928511 | OQ377726 | *Dalbergia sissoo* | Punjab, Pakistan |
| FMB-GHP3-SD | FMB 0148 | MK890312 | MK816928 | MZ423520 | OQ332718 | MT873576 | MW928512 | OQ377727 | *Dalbergia sissoo* | Punjab, Pakistan |
| FMB-GHP7-SD | FMB 0161 | MK890316 | MK816934 | MZ465366 | OQ332719 | OQ183422 | MW928513 | OQ377728 | *Dalbergia sissoo* | Punjab, Pakistan |
| FMB-FSD-SD | FMB 0167 | MK850855 | MK811134 | MZ59514 | OQ332720 | OQ183419 | MW928514 | OQ377729 | *Dalbergia sissoo* | Punjab, Pakistan |
| FMB-BLCN-02 | FMB 0170 | MK820655 | MK811097 | OQ200443 | OQ332721 | OQ183421 | MW928515 | OQ377730 | *Dalbergia sissoo* | Baluchistan, Pakistan |
| FMB-Nosheroferoz-SD | FMB 0173 | MK850850 | MK811094 | OQ200444 | OQ332722 | MT859310 | MW928516 | OQ377731 | *Dalbergia sissoo* | Sindh, Pakistan |
| FMB-BZ1-SD | FMB 0175 | MK910747 | MK811102 | OQ2004445 | OQ262962 | OQ183420 | MW928517 | OQ377732 | *Dalbergia sissoo* | KPK, Pakistan |
| FMB-PZ1-SD | FMB 0176 | MK850854 | MK811101 | OQ200446 | OQ332723 | MT880732 | MW928518 | OQ377733 | *Dalbergia sissoo* | KPK, Pakistan |
| Ceratocystis diversiconidia | CMW22445 | FJ151440.1 | FJ151474.1 | FJ151452.1 | KJ601571 | KJ601607 | KJ601535 | KJ601643 | *Terminalia ivorensis* | Colombia |
| Ceratocystis diversiconidia | CMW22448 | FJ151441.1 | FJ151475 | FJ151453 | KJ601572 | KJ601608 | KJ601536 | KJ601644 | *T. ivorensis* | Colombia |
| Ceratocystis acaciivora | CMW22564 | EU588657.1 | EU588647 | EU588637.1 | KJ601561 | KJ601597 | KJ601525 | KJ601633 | *Acaciivora mangium* | Indonesia |
| Ceratocystis acaciivora | CMW22563 | EU588656.1 | EU588646 | EU588636.1 | KJ601560 | KJ601596 | KJ601524 | KJ601632 | *Acaciivora mangium* | Indonesia |
| Ceratocystis colombiana | CMW5751 | AY177233.1 | EU241493.1 | AY177225.1 | KJ601567 | KJ601603 | KJ601531 | KJ601639 | *Coffea arabica* | Colombia |
| Ceratocystis colombiana | CMW5761 | AY177234.1 | EU241492 | AY177224.1 | KJ601568 | KJ601604 | KJ601532 | KJ601640 | *Coffea arabica* | Colombia |
| Ceratocystis fimbriata | CMW1547 | AF264904 | EF070395.1 | EF070443.1 | KJ601577 | KJ601613 | KJ601541 | KJ601649 | *Imopoea batatas* | Papua New Guinea |
| Ceratocystis fimbriata | CMW14799 | KC493160 | KJ631109 | KC302689 | KJ601578 | KJ601614 | KJ601542 | KJ601650 | *I. batatas* | California, USA |
| Ceratocystis eucalypticola | CMW10000 | FJ236722.1 | FJ236752 | FJ236782.1 | KJ601575 | KJ601611 | KJ601539 | KJ601647 | *Eucalyptus grandis* | South Africa |
| Ceratocystis eucalypticola | CMW11536 | FJ236723.1 | FJ236753.1 | FJ236783.1 | KJ601576 | KJ601612 | KJ601540 | KJ601648 | *E. grandis* | South Africa |
| Ceratocystis adelpha | CMW14809 | DQ520637 | KJ601516 | KJ601509 | KJ601563 | KJ601599 | KJ601527 | KJ601635 | *T. cacao* | Ecuador |
| Ceratocystis adelpha | CMW15051 | AY157951 | KJ601517 | KJ601510 | KJ601564 | KJ601600 | KJ601528 | KJ601636 | *T. cacao* | Costa Rica |
| Ceratocystis mangivora | CMW15052 | EF433298.1 | EF433315 | EF433306 | KJ601586 | KJ601622 | KJ601550 | KJ601658 | *M. indica* | Brazil |
| Ceratocystis mangivora | CMW27305 | FJ200262 | FJ200288 | FJ200275 | KJ601587 | KJ601623 | KJ601551 | KJ601659 | *M. indica* | Brazil |
| Ceratocystis fimbriatomima | CMW24174 | EF190963.1 | EF190957 | EF190951.1 | KJ601579 | KJ601615 | KJ601543 | KJ601651 | *Eucalyptus sp* | Venezuela |
| Ceratocystis fimbriatomima | CMW24377 | EF190966.1 | KJ601520 | EF190954.1 | KJ601581 | KJ601617 | KJ601545 | KJ601653 | *Eucalyptus sp* | Venezuela |
| Ceratocystis fimbriatomima | CMW24176 | EF190964.1 | EF190958 | EF190952.1 | KJ601580 | KJ601616 | KJ601544 | KJ601652 | *Eucalyptus sp* | Venezuela |
| Ceratocystis platani | CMW23450 | KJ631107 | KJ601521 | KJ601513 | KJ601593 | KJ601629 | KJ601557 | KJ601665 | *Platanus orientalis* | Greece |
| Ceratocystis platani | CMW14802 | DQ520630.1 | EF070396 | EF070425.1 | KJ601592 | KJ601628 | KJ601556 | KJ601664 | *Platanus occidentalis* | USA |
| Ceratocystis manginecans | CMW13852 | AY953384 | EF433318 | EF433309.1 | KJ601585 | KJ601621 | KJ601549 | KJ601657 | *Hypocryphalus mangiferae* | Oman |
| Ceratocystis manginecans | CMW13851 | AY953383 | EF433317 | EF433308.1 | KJ601584 | KJ601620 | KJ601548 | KJ601656 | *M. indica* | Oman |
| Ceratocystis mangicola | CMW14797 | AY953382.1 | EF433316 | EF433307.1 | KJ601582 | KJ601618 | KJ601546 | KJ601654 | *M. indica* | Brazil |
| Ceratocystis mangicola | CMW28907 | FJ200257.1 | FJ200283 | FJ200270 | KJ601583 | KJ601619 | KJ601547 | KJ601655 | *Mangifera indica* | Brazil |
| Ceratocystis ecuadoriana | CMW22097 | FJ151434 | FJ151468 | FJ151446.1 | KJ601574 | KJ601610 | KJ601538 | KJ601646 | *E. deglupta* | Colombia |
| Ceratocystis ecuadoriana | CMW22092 | FJ151432.1 | FJ151466 | FJ151444.1 | KJ601573 | KJ601609 | KJ601537 | KJ601645 | *E. deglupta* | Colombia |
| Ceratocystis neglecta | CMW18194 | EF127991.1 | EU881905 | EU881899.1 | KJ601589 | KJ601625 | KJ601553 | KJ601661 | *E. grandis* | Colombia |
| Ceratocystis neglecta | CMW17808 | EF127990.1 | EU881904 | EU881898.1 | KJ601588 | KJ601624 | KJ601552 | KJ601660 | *E. grandis* | Colombia |
| Ceratocystis papillata | CMW10844 | AY177238.1 | EU241481 | AY177229.1 | KJ601591 | KJ601627 | KJ601555 | KJ601663 | *C. arabica* | Colombia |
| Ceratocystis papillata | CMW8856 | AY233867.1 | EU241484 | AY233874 | KJ601590 | KJ601626 | KJ601554 | KJ601662 | *Citrus limon* | Colombia |
| Ceratocystis pirilliformis | CMW6583 | KJ601523 | KJ601522 | KJ601514 | KJ601595 | KJ601631 | KJ601559 | KJ601667 | *E.nitens* | Australia |
| Ceratocystis pirilliformis | CMW6579 | AF427105.1 | AY528983 | DQ371653.1 | KJ601594 | KJ601630 | KJ601558 | KJ601666 | *Eucalyptus nitens* | Australia |
| Ceratocystis cacaofunesta | CMW14798 | AY157952.1 | KJ601518 | KJ601511 | KJ601565 | KJ601601 | KJ601529 | KJ601637 | *T. cacao* | Costa Rica |

*FMB: Fungal Molecular Biology Lab

*CWM is a culture collection number that refers to cultures obtained from the Forestry and Agricultural Biotechnology Institute, University of Pretoria, South Africa

**T. ivorensis* = *Terminalia ivorensis*

**I. batatas = Imopoea batatas*

**E. grandis = Eucalyptus grandis*

**T. cacao* = *Theobroma cacao*

**M. indica = Mangifera indica*

**E. deglupta = Eucalyptus deglupta*

**E. nitens = Eucalyptus nitens*

**C. arabica = Coffea arabica*
